# Supplementary material for: Dynamical analysis and optimal control of the developed information transmission model
Source: PLoS One. 2022 May 23;17(5):e0268326. doi: 10.1371/journal.pone.0268326 (PMC9132490; doi:10.1371/journal.pone.0268326)
Supplement: Attachment — Submitted filename: REVIEW REPORT.pdf [file pone.0268326.s001.pdf]

Review report
PLOS ONE
Manuscript Number: PONE-D-22-05396
Dynamical analysis and optimal control for an information transmission model considering the phenomena of "Super Transmission" and "Asymptomatic Infection"
by
Sida Kang, Xilin Hou, Yuhan Hu, Hongyu Liu

1 Summary and Recommendation:

The authors compared the phenomenon of "Super transmission" and "Asymptomatic infection" in COVID-19 transmission to information transmission. The former is similar to authoritative information transmission individuals, whereas the latter is similar to individuals with low acceptance in information transmission. Also, they constructed an S2EIR model with transmitter authority and individual acceptance levels. Then, they analyzed the asymptotic stability of information-free and information-existence equilibrium on a local and global scale, as well as the model's basic reproduction number, R0. Based on the Pontryagin maximum principle, an optimal control strategy is designed to effectively facilitate information transmission. The numerical simulation corroborates the theoretical analysis results and the system's sensitivity to control parameter changes. This work is well written and the results are new. Before the article can be published, some points need to some minor revision:

1.1 Comments

Following misprints or suggestions are observed. The authors should study the paper carefully for other possible typos.

1) Make sure all equations are properly cited

2) Introductions need to be improved with more details of current work. Please mention which challenges you face to prove the results.

3) What is the difference between stability and asymptotic stability?

4) It is very important to know the advantages of the present study? So, the introduction needs to improve by highlighting the main advantages?.

5) What are the means of the matrixes F and V in equation 6. and how do obtain them? please explain it.

6) How obtain equation 7? add some information.

7) In system 8, replace $S; E_1; E_2; I; R$ with $S^*; E_1^*; E_2^*; I^*; R^*$.

8) How was equation 29 chosen in this form? which conditions followed for it?

9) I suggest to improve the introduction section by studying some useful recent papers/books, for example: https://doi.org/10.1016/j.chaos.2020.109867, https://doi.org/10.1016/j.chaos.2021.110931, https://doi.org/10.1016/j.rinp.2021.104045.

'10) Update references according to the style of journal.

Overall, the work is well written and the results are interesting.

1.2 My Recommendation

I would suggest that the paper should be accepted with minor revision due to some of the corrections I pointed out above and in order to raise the standard of this paper. The English need to be polished, punctuation mark needs to be administered after each equation. Finally, I will be available for further revision of this paper. After the authors take into account the suggestions as above I recommend the publication of the paper.
