# Supplementary material for: Dynamical analysis and optimal control of the developed information transmission model
Source: PLoS One. 2022 May 23;17(5):e0268326. doi: 10.1371/journal.pone.0268326 (PMC9132490; doi:10.1371/journal.pone.0268326)
Supplement: Attachment — Submitted filename: Response to Reviewers.pdf [file pone.0268326.s003.pdf]

Response to Reviewers

Dear Editors and Reviewers:

Thank you very much for your letter and the reviewers' comments concerning our manuscript entitled "Dynamical analysis and optimal control for an information transmission model considering the phenomena of 'Super Transmission' and 'Asymptomatic Infection'" (ID: PONE-D-22-05396). These comments are indeed extremely helpful for revising and improving our paper, as well as the important guiding significance to our researches. We have studied these comments with care and have made correction which we hope meet with approval. The main corrections in the paper and the responds to the reviewer's comments are as flowing:

Responds to the reviewer's comments:

Reviewer #1:

1. Response to comment: (Make sure all equations are properly cited.)

Response: Thank you for helping us to find the errors and omissions. We are very sorry for not being careful to make this obvious mistake. According to your suggestions and requirements, **we have corrected the mistakes cited in the original manuscript. At the same time, according to the whole comments, we have added some equations. Finally, we have checked all equations cited in the manuscript, and then make sure all equations are properly cited in our revised manuscript.**

2. Response to comment: (Introductions need to be improved with more details of current work. Please mention which challenges you face to prove the results.)

Response: Thank you very much for your incisive comments and thorough reminders. I'm very sorry for the inaccurate and unclear expression due to our negligence. According to your comments and suggestions, **we have improved with more details of current work and mentioned the challenges we face to prove the results in our revised manuscript(Page 3, Line 82-99).** The details as following:

"The scholars mentioned above have conducted extensive research on transmitting various types of information via various channels, including the transmission of rumors[4], false information[5], competitive information[28] and malware[27].

Scholars who consider information transmission from the perspective of individuals are usually based on the attitude of transmission individuals to an event[22-24,31,32]. Few scholars consider the transmission individuals' attributes. Generally, most scholars primarily focus on controlling and transmitting negative information in networks[44,46]. On the other hand, confidential information must be transmitted via the contact for information that must be taught in person, such as high technology or practical skills. At the same time, due to the unique characteristics of this type of information, as well as the requirements for the transmitter's authority and the recipient's ability to accept it, there is a shortage of literature on the subject. In addition, the spread of COVID-19 is a hot topic in current research. However, few scholars consider the phenomenon of "Asymptomatic infection" in the transmission model. The research on the transmission of the phenomenon of "Asymptomatic infection" is even less, especially in information transmission. Therefore, consider the phenomenon of "Asymptomatic infection" in the information transmission model and investigate the impact of "Asymptomatic infection" on the information transmission system."

3. Response to comment: (What is the difference between stability and asymptotic stability?)

Response: Thanks for your sincere comments and reminders, which are indeed to the point. Stability and asymptotic stability are important theories in the field of differential equations. It has a very important application in the study of the dynamic system in infectious disease transmission or information transmission. These theories are also applied in our paper. Therefore, we consult some materials to learn the difference between stability and asymptotic stability.

If for any given $\varepsilon > 0$ and $t_0 \geq 0$ all exist $\delta = \delta(\varepsilon, t_0) > 0$, so that as long as x_0 is satisfied:

$$\|x_0 - x_1\| < \delta ,$$

then we can get

$$\|x(t, t_0, x_0) - \varphi(t, t_0, x_1)\| < \varepsilon .$$

For every $t \geq t_0$ establish, then the solution $x = \varphi(t, t_0, x_1)$ of differential equation $\frac{dx}{dt} = f(t, x)$ is said to be stability.

Suppose that $x = \varphi(t, t_0, x_1)$ is stable, and there is $\delta_1 (0 < \delta_1 < \delta)$, so that as long as x_0 satisfies:

$$\|x_0 - x_1\| < \delta_1 ,$$

then we can get

$$\lim_{t \to \infty} \|x(t, t_0, x_0) - \varphi(t, t_0, x_1)\| = 0 .$$

Then the solution $x = \varphi(t, t_0, x_1)$ of differential equation $\frac{dx}{dt} = f(t, x)$ is said to be asymptotic stability.

It can be seen that asymptotic stability is a special case of stability. Asymptotic stability is more stringent than stability for the system. Stability requires that the state trajectory converge to a certain range near the equilibrium point. The asymptotic stability requires that the state trajectory converges to the equilibrium point on the premise of stability. The differential equation constructed in this paper has an equilibrium point, and the system finally converges to the equilibrium point. Therefore, the model in this paper meets the requirements of asymptotic stability. **We think the model constructed in this paper needs the system to reach an asymptotically stability state. Asymptotic stability can better reflect the state of the model. Your comments have prompted us to deepen our understanding of the concepts of stability and asymptotic stability.**

4. Response to comment: (It is very important to know the advantages of the present study? So, the introduction needs to improve by highlighting the main advantages.)

Response: Thanks for your sincere comments and reminders. Your suggestions are of great help to the improvement of the paper. According to your comments and suggestions, **we have improved by highlighting the main advantages in our revised manuscript(Page 3, Line 100-Page 4, Line 119).** The details as following:

"As a result of the above considerations, this paper compares the phenomena of "Super transmission" and "Asymptomatic infection" in COVID-19 transmission to information transmission. It then proposes an *S2EIR* model that incorporates the

phenomena of "Super transmission" and "Asymptomatic infection". During the transmission of COVID-19, the phenomena of "Super transmission" and "Asymptomatic infection" are common. The term "Super transmission" refers to the phenomenon in which highly contagious individuals are more likely to spread the virus to the majority of patients. The term "Asymptomatic infection" refers to a situation in which a patient has been infected with a virus and has become a carrier of the virus but does not exhibit obvious disease symptoms due to individual resistance and physical quality differences. Both of these phenomena frequently occur during information transmission. "Super transmitters" are analogous to authoritative individuals in information transmission. The information transmission by such individuals is more easily accepted. Whereas "Asymptomatic infected individuals" are analogous to hesitant individuals with a low level of acceptance. However, this group of people has certain infectivity in the transmission of COVID-19. However, individuals who conceal information rarely choose to share it with others during the transmission of information. At the same time, others are unaware that such individuals know the information. This is also the manifestation of "Asymptomatic" in information transmission. The optimal control strategy of information transmission is quantified by scientific methods to test these phenomena."

5. Response to comment: (What are the means of the matrixes F and V in equation 6, and how do obtain them? please explain it.)

Response: Thank you very much for your comments! In the original manuscript, perhaps our expression is not comprehensive and careful enough. We would like to make a further explanation on this point.

It is explained in the reference (https://doi.org/10.1016/j.rinp.2021.104045) you recommend, F and V represent the infection and transition matrices respectively. **Meanwhile, we have modified and added the method of obtaining the matrixed F and V in the revised manuscript (Page 6, Line 190-193).** The details as following:

"Calculate the Jacobian matrices of F(X) and V(X) in system (5) respectively, and then take the sub matrices corresponding to the first two variables (i.e. E_1, I) directly related to the number of communicators. The results are as follows:

$$F = \begin{pmatrix} 0 & \alpha(1+m)S \\ \beta & 0 \end{pmatrix}, V = \begin{pmatrix} \beta+\gamma_1+\varepsilon+\mu & 0 \\ 0 & \lambda+\mu \end{pmatrix}, \tag{6}$$

where F and V represent the infection and transition matrices respectively[38]."

6. Response to comment: (How obtain equation 7? add some information.)

Response: Thank you very much for the attentive and earnest comments! We do regret for the lack of smooth logical narration due to the omission of necessary steps and instructions in the original manuscript, which has brought you a dissatisfactory reading experience.

We have modified and added relevant parts in the revised manuscript (Page 6, Line 193-198). The details as following:

(Tip: Due to the addition of equation, equation 7 in the original manuscript has changed to equation 9 in the revised manuscript.)

"By simple calculation, the inverse matrix of V can be obtained as:

$$V^{-1} = \begin{pmatrix} \frac{1}{\beta+\gamma_1+\varepsilon+\mu} & o \\ o & \frac{1}{\lambda+\mu} \end{pmatrix}. \tag{7}$$

The next generation matrix[47] is

$$FV^{-1} = \begin{pmatrix} 0 & \frac{\alpha(1+m)S}{\lambda+\mu} \\ \frac{\beta}{\beta+\gamma_1+\varepsilon+\mu} & 0 \end{pmatrix}. \tag{8}$$

Hence, the basic reproduction number R_0 of system (1) is the spectral radius of the next generation matrix FV^{-1}. Here, the spectral radius is the maximum value of characteristic root of FV^{-1}. Therefore, R_0 can be computed as:

$$R_0 = \rho(FV^{-1}) = \sqrt{\frac{B\alpha\beta(1+m)}{\mu(\beta+\gamma_1+\varepsilon+\mu)(\lambda+\mu)}}. \tag{9}$$"

7. Response to comment: (In system 8, replace S; E_1; E_2; I; R with S^*; E_1^*; E_2^*; I^*; R^*.)

Response: Thank you for helping us to find the omissions. We are very sorry for not being careful to make this obvious mistake. According to your suggestions and requirements, **we have replaced S; E_1; E_2; I; R with S^*; E_1^*; E_2^*; I^*; R^* in system 8 in our revised manuscript (Page 6, Line 203).**

(Tip: Due to the addition of equation, system 8 in the original manuscript has changed to system 10 in the revised manuscript.)

8. Response to comment: (How was equation 29 chosen in this form? which conditions followed for it?)

Response: Thanks for your sincere comments and reminders, which are indeed to the point. According to our understanding， there is no unified standard and rule for constructing Lyapunov function. According to the conditions to be satisfied by Lyapunov function, we have made a lot of attempts. The method we choose just satisfy the conditions of Lyapunov function. Therefore we construct the Lyapunov function as:（Tip: equation 29 has been changed to equation 31 due to the change of equation number. We apologize for the inconvenience.).

$$W(t)=[(S(t)-S^*)+(E_1(t)-E_1^*)+(E_2(t)-E_2^*)+(I(t)-I^*) \\ +(R(t)-R^*)]^2, \tag{31}$$

and

$$\begin{aligned} W'(t) &= 2[(S(t)-S^*)+(E_1(t)-E_1^*)+(E_2(t)-E_2^*)+(I(t)-I^*) \\ &+(R(t)-R^*)][S'(t)+E_1{}'(t)+E_2{}'(t)+I'(t)+R'(t)] \\ &= 2[(S(t)-S^*)+(E_1(t)-E_1^*)+(E_2(t)-E_2^*)+(I(t)-I^*) \\ &+(R(t)-R^*)][B-\mu S-\mu E_1-\mu E_2-\mu I-\mu R]\,. \end{aligned} \tag{32}$$

Because of the existence of $E^*(S^*,E_1^*,E_2^*,I^*,R^*)$, we can know that $B-\mu S^*-\mu E_1{}^*-\mu E_2{}^*-\mu I^*-\mu R^*=0$, so that $B=\mu S^*+\mu E_1{}^*+\mu E_2{}^*+\mu I^*+\mu R^*$. Then, Eq.(32) can be written as:

$$\begin{aligned} W'(t) &= 2[(S(t)-S^*)+(E_1(t)-E_1^*)+(E_2(t)-E_2^*)+(I(t)-I^*) \\ &+(R(t)-R^*)][\mu S^*+\mu E_1{}^*+\mu E_2{}^*+\mu I^*+\mu R^*-\mu S-\mu E_1 \\ &-\mu E_2-\mu I-\mu R] \\ &= -2[(S-S^*)+(E_1-E_1^*)+(E_2-E_2^*)+(I-I^*)+(R-R^*)]^2 \\ &\le 0\,. \end{aligned} \tag{33}$$

Besides that, $W'(t)=0$ holds if and only if $S(t)=S^*,E_1(t)=E_1^*,E_2(t)=E_2^*,I(t)=I^*,R(t)=R^*$. Hence, the information-existence equilibrium point $E^*(S^*,E_1^*,E_2^*,I^*,R^*)$ of system (1) is globally asymptotically stable based on Lyapunov-LaSalle Invariance Principle[48].

We choose a simple and effective way to construct Lyapunov function in this paper. We will try our best to study the construction of Lyapunov function by other methods in the future.

9. Response to comment: (I suggest to improve the introduction section by studying some useful recent papers/books, for example: https://doi.org/10.1016/j.chaos.2020.109867, https://doi.org/10.1016/j.chaos.2021.110931, https://doi.org/10.1016/j.rinp.2021.104045.)

Response: Thanks for your comments and suggestions. We have downloaded and read these references. These references are closely related to the research of this paper, and they deepen our understanding. It is more helpful for us to clearly understand the characteristics of virus transmission and the choice of methods. **Therefore, we have added refers to these useful recent papers in the revised manuscript (Page 2, Line 56-Page 3, Line 67).** The details are as follows:

"Abdo et al. analyzed and found the solution for the model of nonlinear fractional differential equations describing the deadly and most parlous virus, so-called coronavirus (COVID-19). The study discovered that the susceptibility decreases more rapidly at the lower fractional order of the derivative. Similarly, the increase in infections is also rapid, but in a smaller order[37]. Almalahi et al. used fractal-ABC type fractional differential equations by incorporating population self-protection behavior changes to study the dynamics of 2019-nCoV transmission[38], and investigated sufficient conditions of existence and uniqueness of positive solutions for a finite system of φ-Hilfer fractional differential equations[39]. Jeelani et al. investigated a fractional-order mathematical model of COVID-19[40]. These research findings are critical in the prediction of 2019-nCoV."

10. Response to comment: (Update references according to the style of journal.)

Response: Thanks very much for your comments. According to your request, we have checked upon and updated references according to the style of journal. And we have already put them right in the revised version. As these minor errors are more, we have not marked each and every of them corresponding page and line numbers in details.

Special thanks to you for your good comments!

Reviewer #2:

1. Response to comment: (There are some typos and grammatical errors in some parts of this text, especially in the introduction section. Please double-check all sentences and correct all sentences that need to be corrected grammatically.)

Response: Thanks very much for your comments. According to your request, we have checked upon a number of grammatical and typo mistakes once again. And we have already put them right in the revised version. As these minor errors are more, we have not marked each and every of them corresponding page and line numbers in details.

2. Response to comment: (Please pay attention to all punctuation marks in the text.)

Response: Thanks very much for your comments. Your comments are conducive us to be more careful. It is more helpful for us to improve our good scientific research literacy. According to your request, we have checked upon all punctuation once again. And we have already put them right in the revised version. As these minor errors are more, we have not marked each and every of them corresponding page and line numbers in details.

3. Response to comment: (Update the recent references related to this work; Chaos, Solitons & Fractals, 135, 109867. https://doi.org/10.1016/j.chaos.2020.109867; Axioms 2021, 10(3), 228; https://doi.org/10.3390/axioms10030228.)

Response: Thanks for your comments and suggestions. We have downloaded and read these references. These references are closely related to the research of this paper, and they deepen our understanding. It is more helpful for us to clearly understand the characteristics of virus transmission and the choice of methods. **Therefore, we have added refers to these useful recent references in the revised manuscript (Page 2, Line 56-Page 3, Line 67).** The details are as follows:

"Abdo et al. analyzed and found the solution for the model of nonlinear fractional differential equations describing the deadly and most parlous virus, so-called coronavirus (COVID-19). The study discovered that the susceptibility decreases more rapidly at the lower fractional order of the derivative. Similarly, the increase in infections is also rapid, but in a smaller order[37].

Jeelani et al. investigated a fractional-order mathematical model of COVID-19[40]. These research findings are critical in the prediction of 2019-nCoV."

4. Response to comment: (I suggest that the authors amend the article title as follows: "Dynamical analysis and optimal control of the developed information transmission model".)

Response: Thanks for your sincere comments and reminders. Compared with our original title, the title you proposed is more concise and more widely applicable. According to your suggestions and requirements, **we have amended the article title as "Dynamical analysis and optimal control of the developed information transmission model" in our revised manuscript.**

Special thanks to you for your good comments!

We tried our best to improve the manuscript and made some changes in the manuscript. These changes will not influence the content and framework of the paper.

We appreciate for Editors/Reviewers' warm work earnestly, and hope that the correction will meet with approval.

Once again, thank you very much for your comments and suggestions.
