# Supplementary material for: Dynamical analysis and optimal control of the developed information transmission model
Source: PLoS One. 2022 May 23;17(5):e0268326. doi: 10.1371/journal.pone.0268326 (PMC9132490; doi:10.1371/journal.pone.0268326)
Supplement: Attachment — Submitted filename: Report-PONE-D-22-05396.docx [file pone.0268326.s002.docx]

Report on

**Dynamical analysis and optimal control for an information transmission model considering the phenomena of “Super Transmission” and “Asymptomatic Infection”**

**PONE-D-22-05396**

In this paper, the authors compared the phenomenon of “Super transmission” and “Asymptomatic infection” in COVID-19 transmission to information transmission. They then constructed an S2EIR model with transmitter authority and individual acceptance levels and analyzed the asymptotic stability, as well as the model’s basic reproduction number.

Moreover, an optimal control strategy was designed based on the Pontryagin maximum principle. Finally, the numerical simulation is presented.

This paper is very interesting to read. The analysis in this paper is very good. The results are original and present a good degree of novelty. The techniques in this paper present are well-employed to obtain the intended results, and the proofs are correct.

This paper needs a **minor revision**, and I would like to recommend for accepting this paper after the following comments:

1) There are some typos and grammatical errors in some parts of this text, especially in the introduction section. Please double-check all sentences and correct all sentences that need to be corrected grammatically.

2) Please pay attention to all punctuation marks in the text.

3) Update the recent references related to this work; Chaos, Solitons & Fractals, 135, 109867.‏ [*https://doi.org/10.1016/j.chaos.2020.109867*](https://doi.org/10.1016/j.chaos.2020.109867)*;* *Axioms* 2021, *10*(3), 228; <https://doi.org/10.3390/axioms10030228>.

4) I suggest that the authors amend the article title as follows:

"Dynamical analysis and optimal control of the developed information transmission model".
